# Bovine *HOXA11* Gene Identified from *RNA-Seq*: mRNA Profile Analysis and Genetic Variation Detection Using ME Method and Their Associations with Carcass Traits

**DOI:** 10.3390/cells12040539

**Published:** 2023-02-08

**Authors:** Yangming Huang, Kejing Zhang, Yafang Li, Sihuan Zhang, Zhanerke Akhatayeva, Fugui Jiang, Enliang Song, Xianyong Lan

**Affiliations:** 1 College of Animal Science and Technology, Northwest A&F University, Yangling, Xianyang 712100, China; 2 College of Animal Science and Technology, Anhui Agricultural University, Hefei 230036, China; 3 Institute of Animal Science and Veterinary, Shandong Academy of Agriculture Science, Jinan 250100, China

**Keywords:** cattle, *HOXA11*, insertion/deletion (InDel), mathematical expectation (ME), carcass traits, marker-assisted selection (MAS)

## Abstract

The *Homeobox A11* (*HOXA11*) gene regulates limb skeletal development and muscle growth, thus, it was selected as a candidate gene for bovine carcass traits. In this study, we analyzed the mRNA expression level of *HOXA11* in various tissues and cells, and determined the genetic variations in the *HOXA11* gene, which might be used as molecular markers for cattle breeding. The mRNA expression profiles of *HOXA11* in bovine different tissues showed that *HOXA11* was highly expressed in both fat and muscle. The gene expression trend of *HOXA11* in myoblasts and adipocytes indicated that *HOXA11* might be involved in the differentiation of bovine myoblasts and adipocytes. The data in the Ensembl database showed that there are two putative insertion/deletion (InDel) polymorphisms in the bovine *HOXA11* gene. The insertion site (rs515880802) was located in the upstream region (NC_037331.1: g. 68853364-68853365) and named as P1-Ins-4-bp, and the deletion site (rs517582703) was located in the intronic region (NC_037331.1: g. 68859510-68859517) and named as P2-Del-8-bp. These polymorphisms within the *HOXA11* gene were identified and genotyped by PCR amplification, agarose gel electrophoresis and DNA sequencing in the 640 Shandong Black Cattle Genetic Resource (SDBCGR) population. Moreover, the mutation frequency was very low after detection, so the mathematical expectation (ME) method was used for detection. Statistical analysis demonstrated that P1-Ins-4-bp was significantly correlated with the beef shoulder (*p* = 0.012) and tongue root (*p* = 0.004). Meanwhile, P2-Del-8-bp displayed a significant correlation with the back tendon (*p* = 0.008), money tendon (*p* = 2.84 × 10^-4^), thick flank (*p* = 0.034), beef shin (*p* = 9.09 × 10^-7^), triangle thick flank (*p* = 0.04), triangle flank (*p* = 1.00 × 10^-6^), rump (*p* = 0.018) and small tenderloin (*p* = 0.043) in the female SDBCGR population. In summary, these outcomes may provide a new perspective for accelerating the molecular breeding of cattle through marker-assisted selection (MAS) strategies.

## 1. Introduction

With the continuous improvement in human material living standards, the requirements for beef quality, especially for various beef parts, are becoming higher. Therefore, improving carcass traits is a major concern of breeders for profitable beef production [1]. However, although many carcass traits, such as intramuscular fat and the rib-eye area, are moderately or highly hereditary [2], traditional direct selection methods are still inefficient in animal husbandry. Therefore, marker-assisted selection (MAS) strategies [3,4], genome-wide association studies (GWAS), and genome-wide sequencing are increasingly used to study genetic polymorphisms closely related to production traits [5,6]. 

Associations between insertion/deletion (InDel) mutations in promoter and intron regions and carcass traits in cattle have been previously reported. For instance, a 67-bp insertion in the upstream region of *Adiponectin* has been demonstrated to be significantly related to marbling score (MAR) in Hanwoo beef cattle [7]. Similarly, an 18-bp deletion in the eighth intron of *Adenosine monophosphate deaminase 1* has been shown to significantly affect the carcass weight and slaughter weight in Qinchuan cattle [8]. However, research on more precise carcass traits has not been reported so far.

In vertebrates, the homeotic or *HOX* genes, which can be divided into four different *HOX* clusters (*HOXA*, *HOXB*, *HOXC*, and *HOXD*), are distributed on four different chromosomes, encoding 39 *HOX* genes [9,10]. It plays a vital role in regulating almost all basic cellular processes, homeotic transformations and the assignment of morphological traits on each body part along the anterior-posterior (AP) axis [11,12]. The *HOXA* cluster comprises *homeobox A1*, *homeobox A2*, *homeobox A3*, *homeobox A4*, *homeobox A5*, *homeobox A6*, *homeobox A7*, *homeobox A9*, *homeobox A10*, *homeobox A11* (*HOXA11*) and *homeobox A13* [13,14]. *HOXA* cluster genes may be related to the regulation of muscle growth. For instance, *HOXA9* regulates the regenerative ability of satellite cells, providing myonuclei for the growth, hypertrophy, repair and regeneration of postnatal muscle in limbs of aged mice [15,16]. In addition, the lack of *HOXA10* in the satellite cells can lead to genomic instability and cause abnormal chromosome distribution during division, which further leads to the impaired regeneration of hind limb muscles in mice [17]. Furthermore, mutations in the *HOXA* cluster gene also affect muscle growth [18].

Further, many studies have indicated that *HOXA11*, as one of the major genes of the *HOXA* cluster, is associated with limb development, especially in the zeugopod region (radius/ulna and tibia/fibula) in vertebrates [19,20,21]. Early studies have reported that when mice were completely deficient in HOXA11 and HOXD11, the entire bone and muscle loss in the zeugopod region were completely lost [22]. Swinehart et al. (2013) revealed that the absence of *HOXA11* is accompanied by fusion of the extensor digitorum communis and lateralis, the extensor carpi radialis brevis and longus, and the absence of muscle groups, importantly, which is a direct result of the loss of *HOXA11* function rather than the defect of the skeletal model [23]. In addition, the development of limb muscles has derived from satellite cell differentiation, and *HOXA11* seems to regulate the muscle fate of satellite cells during myogenesis through differential expression [24]. Overall, *HOXA11* plays a vital role in modulating muscle development. Muscle characteristics affect many carcass traits. Therefore, we regard it as a candidate gene affecting carcass traits. 

The SDBCGR population is a new cattle breed in China [25], a hybrid of Bohai black cattle, Luxi cattle and Japanese black cattle, with high meat quality [26]. Therefore, this study focused on the SDBCGR population and explored the effects of the genetic variations of *HOXA11* on carcass traits.

## 2. Materials and Methods

### 2.1. Animals’ Welfare

All animal procedures and experiments were permitted by the Animal Policy and Welfare Committee of the Northwest A&F University (protocol No. NWAFAC1008).

### 2.2. Cell Culture

Primary bovine myoblasts were isolated from the longissimus muscle of fetuses (3 to 4 months of development, *n* = 3) [27] and bovine primary adipocytes were isolated from the inguinal fat of fetuses (about 4 to 6 months old, *n* = 3) [28], respectively, according to previous studies in our laboratory [27,28]. Both cells were cultured in a growth medium containing 80% Dulbecco’s modified Eagle’s medium (DMEM, Hyclone; GE Healthcare Life Sciences, Logan, UT, USA) supplemented with 20% fetal bovine serum (FBS; GIBCO, Rockville, MD, USA) and 1% penicillin-streptomycin (Hyclone; GE Healthcare Life Sciences, Logan, UT, USA) in a 5% carbon dioxide incubator at 37 °C. When cells begin to fuse, the growth medium is replaced by the differentiation medium. Myoblasts and adipocytes were cultured in DMEM differentiation medium containing 2% horse serum, 1% penicillin-streptomycin and containing 10% FBS, 10 g/mL insulin (Sigma, I6634, Shanghai, China), 0.5 mmol/L 3-isobutyl-1-methylxanthine (IBMX) (Sigma, I5879, Shanghai, China), 1 mol/L dexamethasone (Sigma, D4902, Shanghai, China) and 1% penicillin-streptomycin, respectively. 

### 2.3. Total RNA Isolation, cDNA Synthesis and Quantitative Real-Time PCR (qRT-PCR)

The spleen, lungs, kidneys, longissimus muscles, visceral fat (perirenal fat) and brain tissues (two males and two females) of four calves were collected from Kingbull Livestock Co., Ltd., (Yangling, Shaanxi, China). Total RNA was isolated from tissue samples and different differentiation stages of myoblasts and adipocytes by Trizol reagent (TaKaRa, Dalian, China), and the RNA was prepared into cDNA (the cDNA was stored at −20 °C) by Prime Script^TM^ RT reagent kit (Takara, Dalian, China) for gene expression profile analysis. Primer pairs for quantitative real-time polymerase chain reaction (qRT-PCR) were designed (Table 1). The 10 µL reaction system contained 5 µL 2 × ChamQ SYBR qPCR Master Mix (Vazyme Biotech Co., Ltd., Nanjing, China), 0.5 µL cDNA, 0.5 µL of each primer, and 3.5 µL of ddH_2_O. The reaction procedure is as follows: pre-denaturation at 95 °C for 30 s; 42 cycles of denaturation at 95 °C for 10 s, annealing and elongation at 60 °C for 30 s. A total of 3 technical replicates were set up for the detection of gene expression levels by qRT-PCR. The relative expression level of genes in tissues was normalized using *GAPDH* and was calculated by the 2^−ΔΔCt^ method [29]. 

### 2.4. Samples and Data Collection

**Figure 1:**
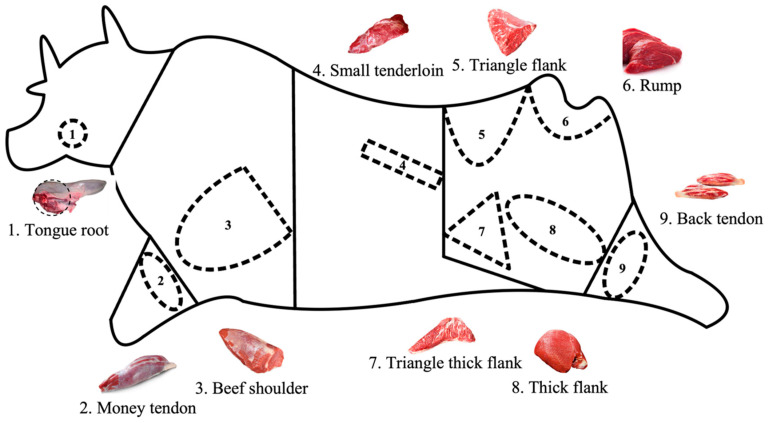
The distribution map of cattle carcass traits involved in this study.

A total of 640 (172 males, 466 females and 2 missing sexes) approximately 30-month-old healthy individuals of the SDBCGR population were randomly selected from two similar farms (Shandong Yangxin Yiliyuan Muslim meat Co., Ltd. and Shandong Kaiyuan animal husbandry Co., Ltd.) (Binzhou and Zhaoyuan, China), then neck muscle tissue samples of each individual were collected. All the healthy individuals had the similar physique and feeding conditions (including feed allocation, environment, and disease control) and were divided into male or female groups. Various carcass traits such as gross weight, left limb weight, etc. (Figure 1) are provided by these companies.

### 2.5. Genomic DNA Isolation, PCR Amplification and Genotyping by ME Method

The phenol–chloroform method was utilized to extract cattle genomic DNA from neck muscle tissues. The specific steps are described clearly in Li’s article [26]. 

The variant table information of *HOXA11* was obtained from the Ensembl database, and two genetic variations were retrieved. Then, based on the reference sequence of bovine *HOXA11* (GenBank accession no. NC_037331.1), two pairs of primers (P1-P2) were designed using NCBI primer blast. (Table 1). A total of 48 individuals were randomly selected for PCR amplification; the PCR reaction volume and amplification steps were the same as described by Huang et al., 2022 [30]. Next, PCR products were detected by 3.5% agarose gel electrophoresis, and the mutation frequencies of both InDel loci were found to be less than 5%, therefore, the genotype of all individual samples was detected by mathematical expectation (ME) method, which is fast and accurate for screening low frequency mutations in large samples [31,32,33]. Moreover, the formula of the ME method has been described in detail in the paper by Yang et al., 2016. The PCR products of each genotype were sequenced by Sangon Biological Technology (Xi’an, China).

### 2.6. Statistical Analysis of Population Genetics

Genotypic frequencies and allelic frequencies, Hardy–Weinberg equilibrium (HWE) and linkage disequilibrium (LD) analyses of the *HOXA11* InDel loci were calculated using the SHEsis platform [34]. Population genetic parameters such as heterozygosity (He), homozygosity (Ho), and the polymorphism information content (PIC) were calculated using the Pop gene (Table 2) [35]. Using SPSS (Version 25.0, IBM, Armonk, NY, USA), the correlation between different genotypes in the cattle *HOXA11* gene and carcass traits was determined by an independent samples *t*-test, and the correlation between different diplotypes of these two loci and carcass traits was determined by one-way ANOVA. *p* < 0.05 was considered significant. A generalized linear model was constructed using the following formula: Y_ij_ = µ + G_i_ + S_j_ + e_ij_, where Y_ij_ is the phenotypic value of carcass traits, µ is the overall population mean, G_i_ is the fixed effect of genotype or combined genotype, S_j_ is the fixed effect of gender, and e_ij_ is the random error [36]. 

## 3. Results

### 3.1. Expression Profiles of HOXA11 in Bovine Tissues, Myoblasts and Adipocytes

**Figure 2:**
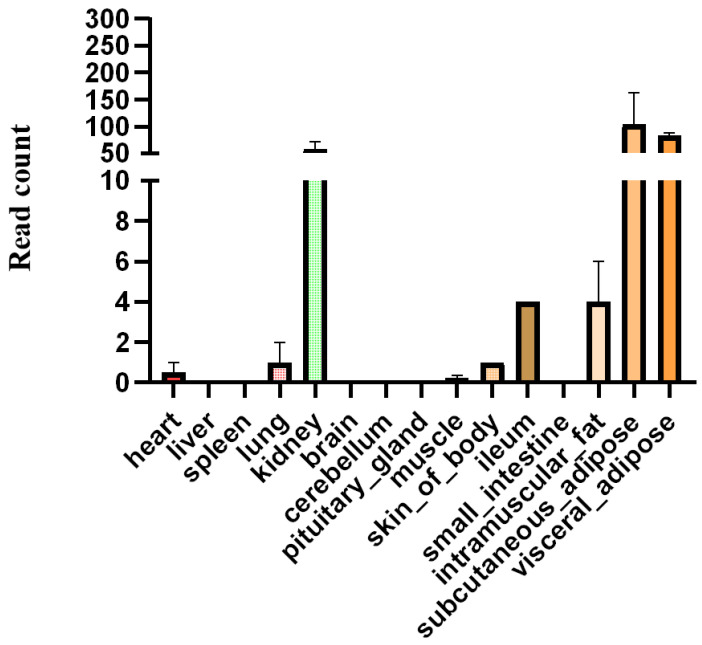
The expression level of *HOXA11* at different tissues of cattle by transcriptome sequencing. Bars represent mean, error bars represent standard error of mean (SEM).

**Figure 3:**
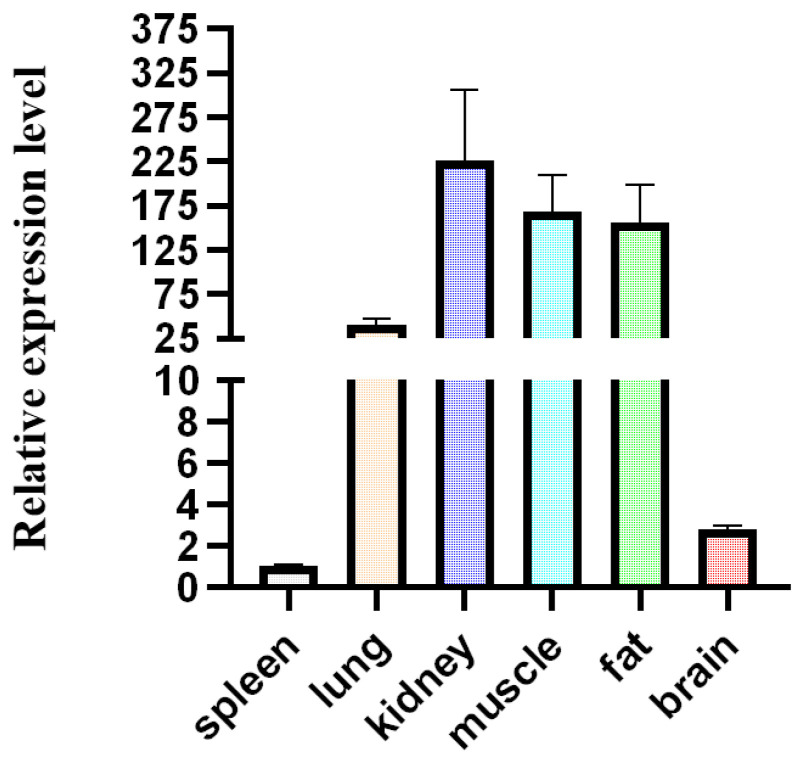
The relative expression level of *HOXA11* at different tissues of calves detected by qRT-PCR. Bars represent mean, error bars represent standard error of mean (SEM).

**Figure 4:**
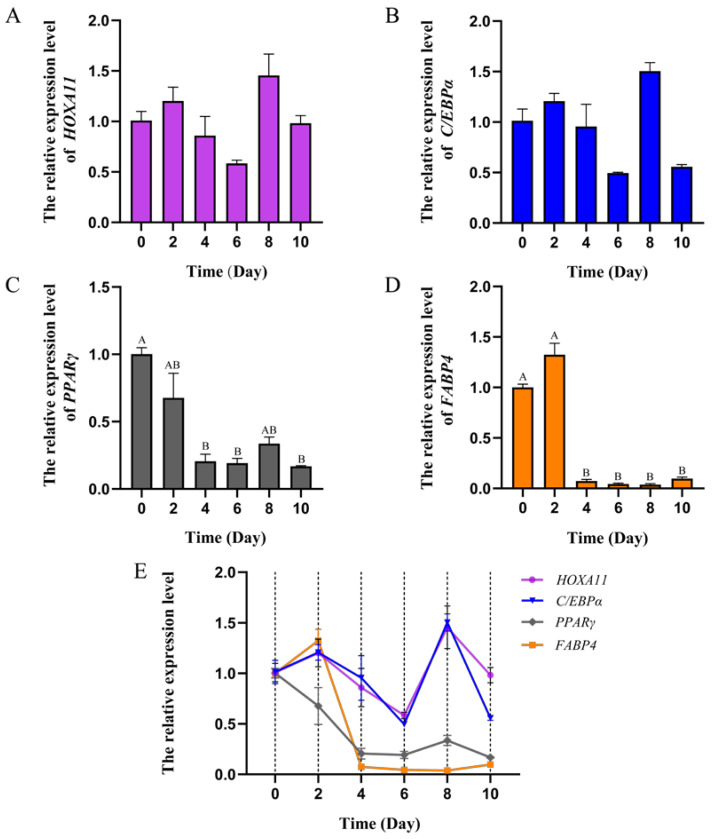
Expression characteristics of *HOXA11* and fat-development-associated genes in bovine adipocytes. Expression trend of *HOXA11* (**A**), *C/EBPα* (**B**), *PPARγ* (**C**) and *FABP4* (**D**) in bovine adipocytes in differentiation medium (0, 2, 4, 6, 8 and 10 days; *n* = 3), and the expression trend line chart of these genes (**E**). The columns with different superscripts (**A**,**B**) within each figure differ significantly at *p* < 0.01 level. Bars represent mean, error bars represent standard error of mean (SEM).

**Figure 5:**
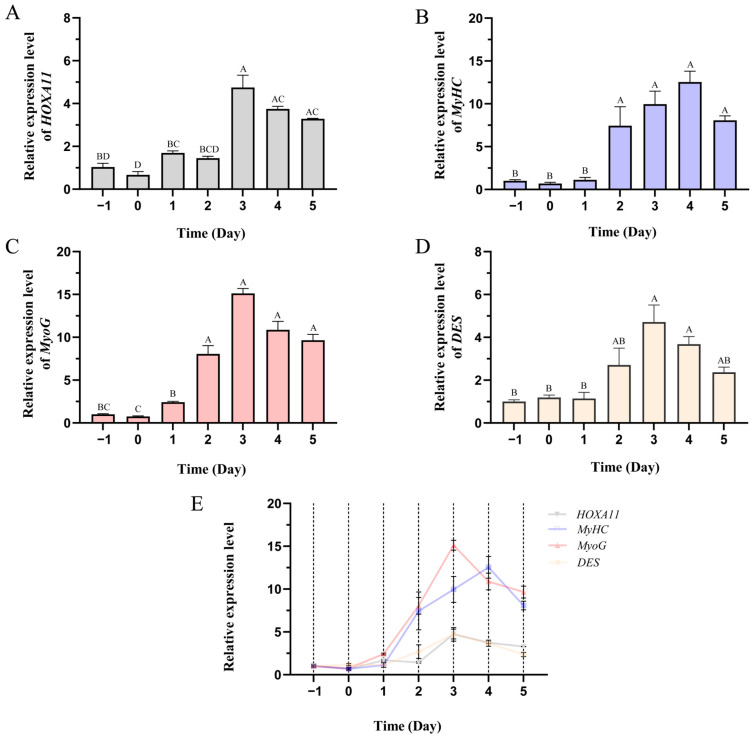
Expression characteristics of *HOXA11* and muscle-development-associated genes in bovine myoblasts. Expression trend of *HOXA11* (**A**), *MyHC* (**B**), *MyoG* (**C**) and *DES* (**D**) in bovine myoblasts cultured in proliferation medium (−1 day) and differentiation medium (0, 1, 2, 3, 4 and 5 days; *n* = 3), and the expression trend line chart of these genes (**E**). The columns with different superscripts (**A**–**D**) within each figure differ significantly at *p* < 0.01 level. Bars represent mean, error bars represent standard error of mean (SEM).

The *HOXA11* gene with high expression in subcutaneous and visceral fat was found in the previous study [37]. According to the previous transcriptome data [37], we found that *HOXA11* is also expressed in other tissues (Figure 2). Due to the important roles of fat and muscle in bovine development and beef quality, in order to reveal the function of *HOXA11*, we studied the expression profiles of *HOXA11* in different tissues of cattle (Figure 3). We found low expression levels of *HOXA11* in the spleen, brain and lung, and high expression levels in the fat and kidney, consistent with transcriptome data [37], but high expression levels of *HOXA11* in skeletal muscle, which contradict transcriptome data (Figure 3). At the cellular aspect, we measured the expression level of *HOXA11*, *C/EBPα*, *PPARγ* and *FABP4* genes at different stages (0, 2, 4, 6, 8 and 10 days; *n* = 3) of adipocyte differentiation (Figure 4), and the expression levels of *HOXA11*, *DES*, *MyHC* and *MyoG* at different stages (−1, 0, 1, 2, 3, 4 and 5 days; *n* = 3) of myoblast differentiation (Figure 5), which were important in the regulation of adipocytes and myoblast, respectively. The expression characteristic of *HOXA11* showed a significant positive correlation with the expression of *C/EBPα* (Pearson’s r = 0.828, *p* = 0.042) (Table 3), *DES* (Pearson’s r = 0.840, *p* = 0.018), *MyHC* (Pearson’s r = 0.863, *p* = 0.012), and *MyoG* (Pearson’s r = 0.913, *p* = 0.004) (Table 4). These results suggested that *HOXA11* might be involved in the development of bovine myoblasts and adipocytes.

### 3.2. Identification of InDels by the ME Method and Sequencing Validation

**Figure 6:**
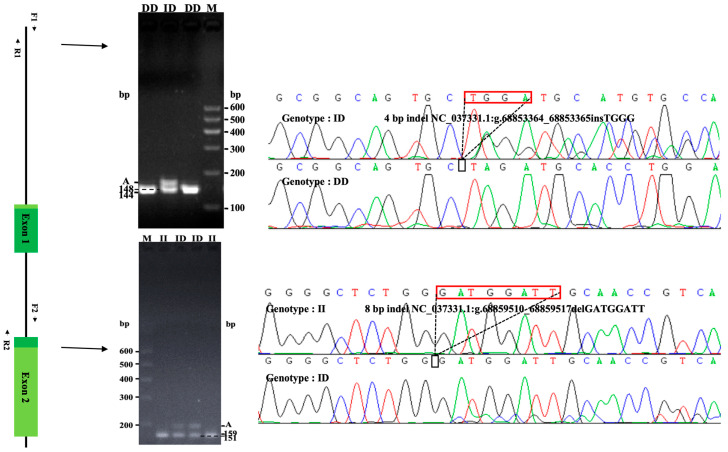
Agarose electrophoresis and sequencing chromas of InDels in the cattle *HOXA11* gene. Agarose electrophoresis and sequencing chromas for cattle *HOXA11* P1-Ins-4-bp InDel. Agarose electrophoresis and sequencing chromas for cattle *HOXA11* P2-Del-8-bp InDel. Sequencing chromas showed homozygous insertion type (II), heterozygous genotype (ID) and homozygous deletion type (DD). The A represents the non-target fragment, which is heteroduplex.

After testing 48 random DNA samples, the electrophoresis pattern and sequencing map showed that both mutation sites within the *HOXA11* gene are polymorphic, which were detected at 3953-bp upstream (NC_037331.1: g. 68853364-68853365), named as P1-Ins-4-bp, and intron 1 (NC_037331.1: g. 68859510-68859517), named as P2-Del-8-bp (Table 1). Both mutations were only present in the homozygous reference or heterozygous states for the sample tested. The P1-Ins-4-bp locus had the homozygous deletion (DD) and heterozygous genotype (ID), while the P2-Del-8-bp locus had the homozygous insertion (II) and heterozygous genotype (ID) (Figure 6). Statistical analysis showed that the mutation frequency was less than 5%, so we decided to use the ME method for subsequent experiments.

**Figure 7:**
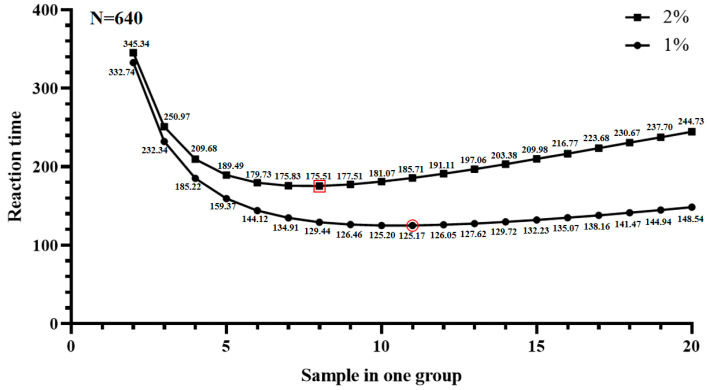
The reaction times of different sizes of one mixed group. The least reaction time will be obtained when the estimated mutation frequencies are 1% and 2%, and the sample sizes in one group are 11 and 8, respectively.

According to the estimated mutation frequency and the equation obtained by the ME method, the optimal number of individuals in one mixed group (NGn) was 8 (P1-Ins-4-bp) and 11 (P2-Del-8-bp) (Figure 7, Table 5). The predicted reaction times of P1-Ins-4-bp and P2-Del-8-bp by formula were 176 and 126, respectively. It has been shown that the actual reaction times (RT) depend on the presence of a single band in a mixed group consisting of different cattle. However, when detecting the P1-Ins-4-bp locus with the ME method, we found false positive phenomena with two bands in all mixed groups, but when we tested with a single sample, there were no false-positive phenomena; this may be caused by some problems with the primer itself or contamination. Therefore, the P1-Ins-4-bp locus was detected with a single sample, the P2-Del-8-bp locus was detected via the ME method. The reaction times of P2-Del-8-bp were counted as 221. Compared with the traditional detection method, the PCR times of P2-Del-8-bp were decreased by 65.47% using the ME method.

### 3.3. Genotypic Frequencies and Population Indices

The frequency of the DD genotype (0.897) was higher than the ID genotype (0.103) within the P1-Ins-4-bp locus. Similarly, for the P2-Del-8-bp locus, the frequency of II genotype was higher (0.981). In addition, both mutation sites identified in *HOXA11* conformed to the HWE (*p* > 0.05). Moreover, the PIC value showed that the two detected *HOXA11* mutations in the SDBCGR population were characterized as low polymorphic (0 < PIC ≤ 0.25) (Table 2).

### 3.4. Linkage Disequilibrium (LD) and Haplotype Analyses

**Figure 8:**
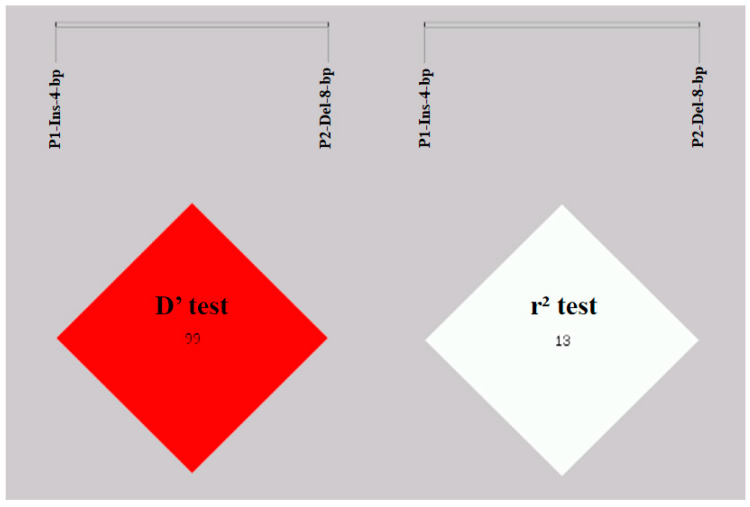
Linkage equilibrium analysis of P1-Ins-4-bp and P2-Del-8-bp in the SDBCGR population.

**Figure 9:**
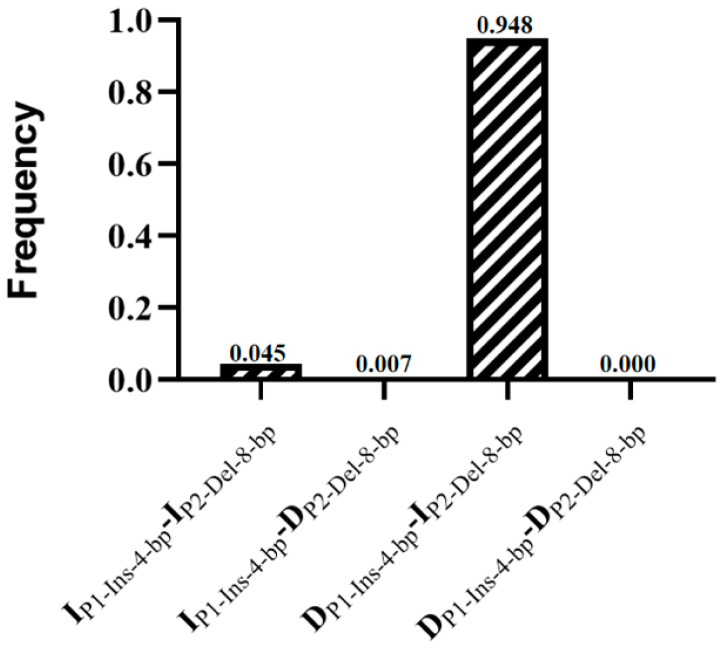
Haplotype frequencies of the *HOXA11* gene in the SDBCGR population.

To further explore whether there is a linkage between these two InDel loci of *HOXA11*, we performed LD analysis using the SHEsis online platform. The results showed that the values for D’ and r^2^ were 1.00 and 0.13, respectively, indicating that there was not strong linkage between P1-Ins-4-bp and P2-Del-8-bp (Figure 8). The haplotype analysis results for *HOXA11* revealed four haplotypes, and DP1-Ins-4-bp-IP2-Del-8-bp had the highest frequency (Figure 9).

### 3.5. Association Analysis between HOXA11 InDels/Diplotypes and Carcass Traits

**Figure 10:**
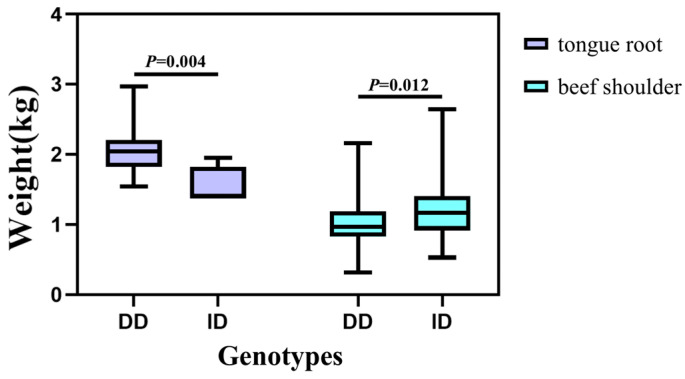
Association of the P1-Ins-4-bp InDel with carcass traits in the female SDBCGR population.

**Figure 11:**
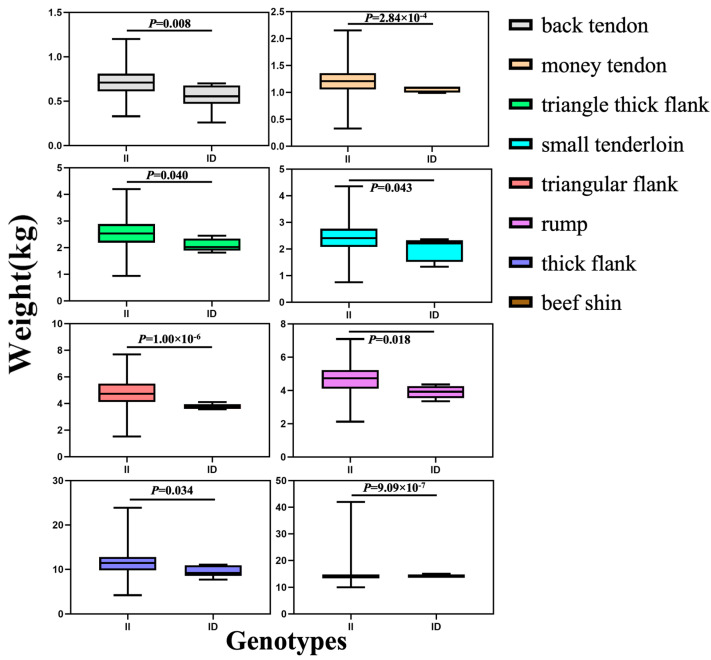
Association of the P2-Del-8-bp InDel with carcass traits in the female SDBCGR population.

**Figure 12:**
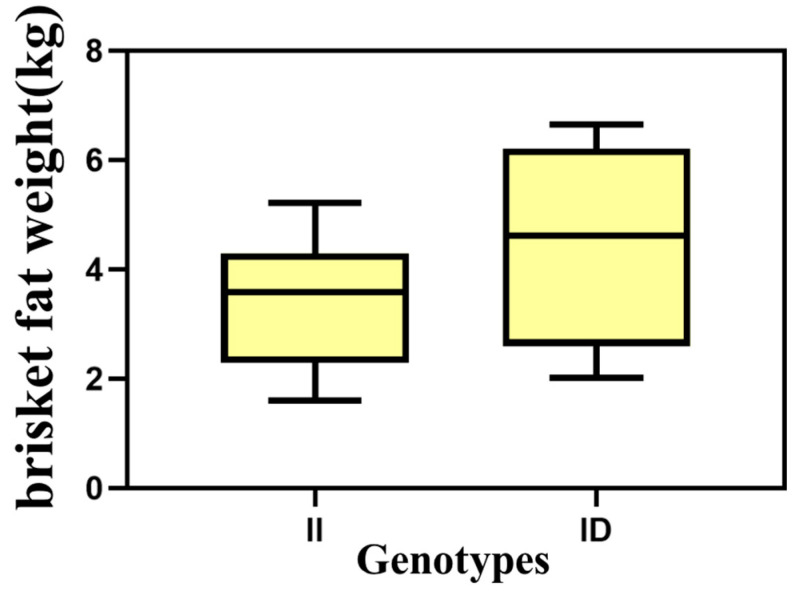
Association of the P2-Del-8-bp InDel with carcass traits in the male SDBCGR population.

**Figure 13:**
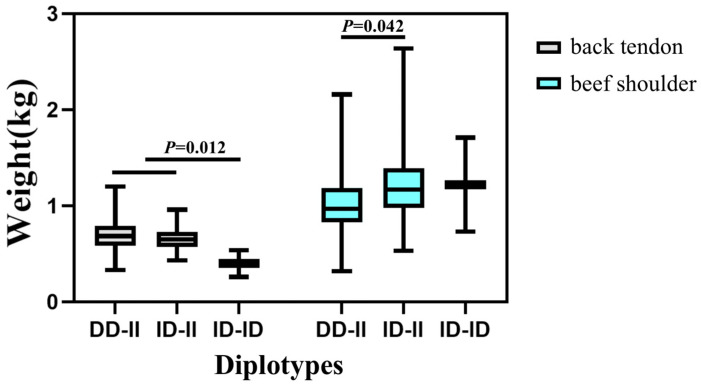
Association of diplotypes with carcass traits in the female SDBCGR population.

The association analysis between two InDel loci in the *HOXA11* gene and more than 50 carcass traits has been studied in different genders (172 males and 466 females) of the SDBCGR population. Significant associations were observed between the P1-Ins-4-bp locus in the *HOXA11* gene and beef shoulder (*p* = 0.012) and tongue root (*p* = 0.004) in the female SDBCGR population (Table 6), whereas no significant associations were observed for males. For females, individuals with the heterozygous genotype had a better beef shoulder phenotype than individuals with the homozygous genotype; however, the opposite was true for the tongue root phenotype. (*p* < 0.05; Table 6, Figure 10). Therefore, which genotype is more favorable depends on the specific breeding situation. In addition, the P2-Del-8-bp locus in the *HOXA11* gene was significantly associated with back tendon (*p* = 0.008), money tendon (*p* = 2.84 × 10^−4^), thick flank (*p* = 0.034), beef shin (*p* = 9.09 × 10^−7^), triangle thick flank (*p* = 0.04), triangle flank (*p* = 1.00 × 10^−6^), rump (*p* = 0.018) and small tenderloin (*p* = 0.043) in the female SDBCGR population. Importantly, individuals with the homozygous genotype had a superior phenotype than individuals with the heterozygous genotype. Furthermore, for males, the brisket fat of individuals with the heterozygous genotype was the dominant genotype (*p* < 0.05; Table 7, Figure 11 and Figure 12). Additionally, in the diplotype analysis, individuals with ID-II diplotypes had a better beef shoulder phenotype than individuals with DD-II diplotypes in females (*p* < 0.05) (Table 8, Figure 13). However, for the carcass traits of the male SDBCGR population, no significant difference was found between diplotypes.

## 4. Discussion

In this study, polymorphisms at the upstream P1-Ins-4-bp locus and the intron P2-Del-8-bp locus of the *HOXA11* gene were detected in association with SDBCGR bovine carcass traits (beef shoulder, tongue root, back tendon, money tendon, thick flank, beef shin, triangle thick flank, triangle flank, rump, etc.). Moreover, for the P2-Del-8-bp locus, we adopted the ME method for detection. Compared with the traditional method for single detection of a large number of samples, it not only saves time and money, but is easy to operate. In our laboratory, we have previously used this method to detect polymorphism in large samples of cattle and sheep [31,32,33]. In this study, compared with traditional methods, the number of responses required for the accuracy of the ME strategy was reduced to 221 times (SDBCGR, *n* = 640), making the ME strategy simpler and more effective. Furthermore, our results revealed that the P1-Ins-4-bp locus and P2-Del-8-bp locus of the cattle *HOXA11* gene were present in HWE in SDBCGR (*p* > 0.05).

More interestingly, in our study, almost all significant carcass traits were derived from females. Previous studies showed that HOXA11 itself can determine the transcription of *Prolactin (PRL)* gene in endometrial stromal cells, and there is an interaction between HOXA11 and FOXO1. When HOXA11 binds to FOXO1, it can also regulate the up-regulation of IGFBP-1 [38,39]. Therefore, we speculated that HOXA11 can affect body growth by regulating hormone secretion in animals.

Previous studies have shown that *HOXA11* is essential for the regulation of limb skeletal development, especially of the zeugopod region [19,40]. Interestingly, the carcass traits of the limbs involved in our study were the back tendon and money tendon. On the one hand, during embryonic limb development, *HOXA11* may regulate the migration and aggregation as well as precursor cell differentiation through expression in limb muscle precursor and mesenchymal cells, respectively, thereby affecting limb muscle shape and arrangement. It has been reported that the HOXA11 protein is expressed in the muscle precursor cells from the dermomyotomal compartment invading the wing bud at stage 19, and this expression is caused by the induced interaction of the limb mesenchyme [41]. Subsequently, as the muscle precursor cells migrate to the wing buds and aggregate into dorsal and ventral muscle masses, the level of HOXA11 protein in the muscle mass gradually decreases until it is no longer detectable in the muscle mass at stage 26 [42]. This indicates that during the early limb bud formation, the *HOXA11* gene in muscle precursor cells activated by mesenchymal cells might directly affect the migration and accumulation of muscle precursor cells in limb buds, but it no longer directly affects muscle precursor cells after the formation of muscle mass. In addition, when muscle precursor cells assemble and migrate to form dorsal and ventral muscle masses, different muscle precursor population tissues will differentiate to form different muscle bundles; *HOXA11* expressed in mesenchyme may change the microenvironment of muscle precursor cells by regulating the expression patterns of extracellular matrix around muscle precursors, thereby indirectly affecting the migration, proliferation and differentiation of muscle precursors. Studies have shown that *HOXA11* is expressed in muscle connective tissues and tendons in the zeugopod region of the mouse forelimb, but not in muscles, and that *HOXA11*/*HOXD11* double mutant mice are accompanied by fusion between muscles, and the absence or disorganization of muscle groups and tendons. More importantly, this is not a secondary effect due to defects in skeletal patterning, but a direct result of the loss of the *HOXA11* function [23]. Moreover, *HOXA11* knockdown in the uterosacral ligaments increases the degradation of the extracellular matrix [43]. Since connective tissues and ligaments are derived from embryonic mesenchymal cells, one possible mechanism is that during embryonic limb development, the mutation of *HOXA11* may directly regulate the migration and aggregation of precursor cells by regulating its expression in limb muscle precursor cells, as well as indirectly regulate the migration, proliferation and differentiation of precursor cells by regulating its expression in mesenchymal cells, thus, affecting the shape and assemble of limb muscles and resulting in changes in muscle weight in different parts of the limb. 

On the other hand, after birth, *HOXA11* may affect the proliferation of muscle satellite cells in the limbs, which in turn affects postnatal muscle growth and maintains its hypertrophy, thereby affecting the shape of postnatal adult muscles. Both the *HOXA10* gene and *HOXA11* are highly expressed in mouse limb muscle satellite cells and the lack of the *HOXA10* gene in muscle satellite cells can result in genomic instability caused by abnormal chromosome distribution during the division of muscle stem cells, leading to stagnation of muscle satellite cell proliferation and hind limb muscle regeneration disorders [17]. The rapid hypertrophy of muscle fibers in the initial phase of muscle growth is due to the provision of muscle nuclei with satellite cells between birth and three weeks after birth [44]. In addition, after three weeks of birth, the addition of muscle nuclei from satellite cells is indispensable for adult muscle hypertrophy [45]. Therefore, another potential mechanism is that after embryo birth, *HOXA11* may regulate the proliferation and regeneration capacity of muscle satellite cells by regulating their expression, thereby affecting the growth and hypertrophy of muscle after the birth of an embryo, and further influencing the shape and assembling of limb muscles to change the muscle weight in different parts of the limb. Therefore, we believe that the *HOXA11* gene can promote muscle growth. However, specific investigations on how the *HOXA11* gene mutation regulates the muscle weight in different parts of the bovine body require further study.

## 5. Conclusions

In conclusion, in this study, we found that the *HOXA11* gene was highly expressed in muscle and fat tissues, indicating that it might be involved in the regulation of muscle and fat development. Then, we found that two InDel variations of the *HOXA11* gene were significantly correlated with the carcass traits of SDBCGR population. Our results can be used in future cattle breeding strategies based on MAS to improve the economic efficiency of the cattle industry.

## Figures and Tables

**Table 1:** Primers used in this study.

Primer Names	Primer Pairs (5′-3′)	Sizes (bp)	Location	Note
P1-Ins-4-bp	F:ACTGACCATGCCAAGGCTAC	148/144	Upstream	rs515880802
	R:TTGAGCTCTGCACTCCACTC			
P2-Del-8-bp	F:TAGTCGGGGGACCTTGCTTG	159/151	Intron 1	rs517582703
	R:GCTTCTTTCGGGTTCGTTGG			
MyoG	F:CCAGTGAATGCAGCTCCCATA	88	Exon 2–3	qRT-PCR
	R:AGCAGATGATCCCCTGGGTTG			
MyHC	F:TGCTCATCTCACCAAGTTCC	150	Exon 41–42	qRT-PCR
	R:CACTCTTCACTCTCATGGACC			
DES	F:AACAATTTGGCTGCCTTCCG	97	Exon 2–3	qRT-PCR
	R:ACGCGATTTCCTCGTTGAGA			
C/EBPα	F:TGGACAAGAACAGCAACGAG	130	Exon 1	qRT-PCR
	R:TTGTCACTGGTCAGCTCCAG			
PPARγ	F:AGGATGGGGTCCTCATATCC	121	Exon 6	qRT-PCR
	R:GCGTTGAACTTCACAGCAAA			
FABP4	F:AAGTCAAGAGCATCGTAA	111	Exon 2–3	qRT-PCR
	R:CCAGCACCATCTTATCAT			
HOXA11	F:GGCCACACTGAGGACAAG	144	Exon 1–2	qRT-PCR
	R:TAGTTGCAGGCGTTTCTCTT			
GAPDH	F:ACCACTTTGGCATCGTGGAG	76	Exon 7–8	qRT-PCR
	R:GGGCCATCCACAGTCTTCTG			

**Table 2:** Genetic parameters of InDels within the *HOXA11* gene in SDBCGR population.

Loci	Sizes	Genotypic Frequencies			Allelic Frequencies		HWE	Population Parameters			
	N	II	ID	DD	I	D	*p*-Value	*Ho*	*He*	*Ne*	PIC
P1-Ins-4-bp	416	0	0.103	0.897	0.052	0.948	*p* > 0.05	0.902	0.098	1.109	0.093
P2-Del-8-bp	640	0.981	0.019	0	0.991	0.009	*p* > 0.05	0.981	0.019	1.019	0.018

Note: **HWE**, Hardy–Weinberg equilibrium; ***Ho***, homozygosity; ***He***, heterozygosity; ***Ne***, effective allele numbers; **PIC**, Polymorphism information content.

**Table 3:** Pearson correlation analyses between the expression of *HOXA11* and fat-development-associated genes in differentiation of adipocytes.

Gene	Pearson’s r	Sig. (2-Tailed)
*C/EBPα*	0.828 *	0.042
*PPARγ*	0.475	0.341
*FABP4*	0.288	0.580

Note: * *p* < 0.05.

**Table 4:** Pearson correlation analyses between the expression of *HOXA11* and muscle-development-associated genes in differentiation of muscle cells.

Gene	Pearson’s r	Sig. (2-Tailed)
*DES*	0.840 *	0.018
*MyHC*	0.863 *	0.012
*MyoG*	0.913 **	0.004

Note: * *p* < 0.05; ** *p* < 0.01.

**Table 5:** The reaction times of different loci in the SDBCGR population.

Types	P1-Ins-4-bp	P2-Del-8-bp
Sample sizes	640	640
Estimated mutation frequency	0.02	0.01
NR_1_ (number of individuals in one reaction time)	1	1
RT_1_ (reaction times)	640	640
NG_n_ (the optimal number of individuals in one mixed group)	8	11
pRT_n_ (predicted reaction times by the formulate)	176	126
pRR (predicted reduction rate)	72.50%	80.31%
RT_n_ (reaction times)	-	221
RR (reduction rate)	-	65.47%

**Table 6:** Associations of the P1-Ins-4-bp InDel with carcass traits in the female SDBCGR population.

Carcass Traits	Observed Genotypes (Mean ± SE)		*p* Values
	ID	II	
Beef shoulder (kg)	1.30 ^a^ ± 0.1 (*n* = 29)	1.02 ^b^ ± 0.02 (*n* = 163)	0.012
Tongue root (kg)	1.53 ^B^ ± 0.14 (*n* = 4)	2.02 ^A^ ± 0.05 (*n* = 29)	0.004

Note: Numbers with different letters (a, b) means *p* < 0.05, (A, B) means *p* < 0.01.

**Table 7:** Associations of the P2-Del-8-bp InDel with carcass traits in different genders of the SDBCGR population.

Carcass Traits	Observed Genotypes (Mean ± SE)		*p* Values
	ID	II	
**Female**			
Back tendon (kg)	0.55 ^B^ ± 0.06 (*n* = 6)	0.71 ^A^ ± 0.01 (*n* = 363)	0.008
Money tendon (kg)	1.06 ^B^ ± 0.02 (*n* = 6)	1.21 ^A^ ± 0.01 (*n* = 366)	2.84 × 10^−4^
Thick flank (kg)	9.47 ^b^ ± 0.53 (*n* = 6)	11.37 ^a^ ± 0.11 (*n* = 363)	0.034
Beef shin (kg)	14.17 ^B^ ± 0.17 (*n* = 6)	15.92 ^A^ ± 0.28 (*n* = 323)	9.09 × 10^−7^
Triangle thick flank (kg)	2.09 ^b^ ± 0.1 (*n* = 6)	2.53 ^a^ ± 0.03 (*n* = 367)	0.040
Triangle flank (kg)	3.79 ^B^ ± 0.08 (*n* = 6)	4.78 ^A^ ± 0.05 (*n* = 367)	1.00 × 10^−6^
Rump (kg)	3.90 ^b^ ± 0.15 (*n* = 6)	4.73 ^a^ ± 0.04 (*n* = 365)	0.018
Small tenderloin (kg)	2.00 ^b^ ± 0.18 (*n* = 6)	2.43 ^a^ ± 0.03 (*n* = 358)	0.043
**Male**			
Brisket fat (kg)	4.48 ^a^ ± 0.96 (*n* = 4)	3.36 ^b^ ± 0.11 (*n* = 82)	0.045

Note: Numbers with different letters (a, b) means *p* < 0.05, (A, B) means *p* < 0.01.

**Table 8:** Associations of different diplotypes of the P1-Ins-4-bp and P2-Del-8-bp InDels with carcass traits in the female SDBCGR population.

Carcass Traits	Observed Diplotypes (Mean ± SE)				*p* Values
	DD-II	DD-ID	ID-II	ID-ID	
Beef shoulder (kg)	1.02 ^b^ ± 0.02 (*n* = 163)	-	1.3 ^a^ ± 0.11 (*n* = 27)	(*n* = 2)	0.042

Note: Numbers with different letters (a, b) means *p* < 0.05.

## Data Availability

Data are available upon request from corresponding author.
